# Genome-Wide Identification and Characterization of Long Non-Coding RNAs from Mulberry (*Morus notabilis*) RNA-*seq* Data

**DOI:** 10.3390/genes7030011

**Published:** 2016-02-29

**Authors:** Xiaobo Song, Liang Sun, Haitao Luo, Qingguo Ma, Yi Zhao, Dong Pei

**Affiliations:** 1 College of Biological Sciences and Technology, Beijing Forestry University, Beijing 100083, China; songxiaoboo@126.com; 2 State Key Laboratory of Tree Genetics and Breeding, Research Institute of Forestry, Chinese Academy of Forestry, Beijing 100091, China; maqgme@163.com; 3 Key Laboratory of Intelligent Information Processing, Institute of Computing Technology, Chinese Academy of Sciences, Beijing 100190, China; luohaitao@ict.ac.cn (H.L.); biozy@ict.ac.cn (Y.Z.); 4 Precision Medicine Center, Research Institute of Information Industry for LuoYang (LuoYang Branch of Institute of Computing Technology, Chinese Academy of sciences), Luoyang 471000, China

**Keywords:** lncRNAs, *Morus notabilis*, RNA-*seq*

## Abstract

Numerous sources of evidence suggest that most of the eukaryotic genome is transcribed into protein-coding mRNAs and also into a large number of non-coding RNAs (ncRNAs). Long ncRNAs (lncRNAs), a group consisting of ncRNAs longer than 200 nucleotides, have been found to play critical roles in transcriptional, post-transcriptional, and epigenetic gene regulation across all kingdoms of life. However, lncRNAs and their regulatory roles remain poorly characterized in plants, especially in woody plants. In this paper, we used a computational approach to identify novel lncRNAs from a published RNA-*seq* data set and analyzed their sequences and expression patterns. In total, 1133 novel lncRNAs were identified in mulberry, and 106 of these lncRNAs displayed a predominant tissue-specific expression in the five major tissues investigated. Additionally, functional predictions revealed that tissue-specific lncRNAs adjacent to protein-coding genes might play important regulatory roles in the development of floral organ and root in mulberry. The pipeline used in this study would be useful for the identification of lncRNAs obtained from other deep sequencing data. Furthermore, the predicted lncRNAs would be beneficial towards an understanding of the variations in gene expression in plants.

## 1. Introduction

Mulberry (*Morus notabilis*) belongs to the genus *Morus*, which comprises 10–13 species and over 1000 cultivars distributed throughout Asia, Africa, Europe, and North America [1,2], and are well known for their important economic and medicinal values [3]. In China, mulberry leaves have been used to feed silkworms for silk production [4], and its fruit is either eaten fresh or widely used in the production of juice, wine, jam and canned food [5]. In addition, the root, bark, branch, leaf, and fruit of mulberry have been used for protecting liver, improving eyesight, treating fever, facilitating urination, and lowering blood pressure due to their high levels of isoprenylated flavonoids, such as sanggenon-type flavanones, Diels-Alder adducts, and flavones [6,7,8]. Previous studies have suggested that secondary metabolism products and some small molecule modulators might play critical roles in plant-herbivore interactions, and mulberry is an ideal research model organism used to study plant-herbivore interaction [9,10]. The genome sequencing of *Morus notabilis* was completed in 2013, with approximately 29,338 protein-coding genes identified, however, a lot of important information has not been exploited completely [10,11]. Therefore, it is necessary and urgent to identify novel lncRNAs and understand the functions of lncRNAs in *Morus notabilis*.

Recent advances in DNA sequencing technology and transcriptome analysis have challenged the central dogma of biology. Emerging evidence shows that more than 90% of eukaryotic genomes are transcribed, but only 1%–2% have a protein-coding capacity, and the majority of sequences are transcribed as noncoding RNAs (ncRNAs) [12,13], which play critical roles in regulating gene expression at the transcriptional, post-transcriptional, and epigenetic levels during several biological processes [14,15,16]. Based on their distinct characteristics compared to housekeeping ncRNAs, including rRNAs, tRNAs, and small nucleolar RNAs, ncRNAs can be classified as (1) small RNAs, including microRNAs (miRNAs) and small interfering RNAs (siRNAs); (2) natural antisense transcripts (NATs); and (3) long non-coding RNAs (lncRNAs) [17]. LncRNAs have been defined as non-protein coding RNAs of more than 200 bp in length, distinguishing them from short ncRNAs [18,19].

Since the first report of lncRNAs in humans [20], thousands of lncRNAs have been identified in a number of species. However, genome-wide identifications of lncRNAs have been performed in only a few plant species [17,21]. For instance, vernalization in Arabidopsis is influenced by the lncRNAs COOLAIR and COLDAIR [22,23] and induced by phosphate starvation1 (IPS1), which is a member of the TPS1/Mt4 gene family that acts as a miR399 target mimic in fine tuning of PHO2 (encoding an E2 ubiquitin conjugase-related enzyme) expression and phosphate uptake in Arabidopsis, tomato and *Medicago truncatula* [24,25]. A large set of *Populus* RNA-*seq* data was examined and a total of 504 lncRNAs were found to be drought responsive [26]. A network of interactions among the lncRNAs, miRNAs and mRNAs was constructed with the RNA-*seq* data of *Populu stomentosa*, revealing that lncRNAs were involved in the regulation of wood formation [27]. Each of the lncRNA surveys in plants uncovered a substantial number of lncRNAs, which were often expressed at low levels in a tissue-specific manner, as in humans and other mammals, and acted as natural miRNA target mimics, chromatin modifiers, or molecular cargo for protein re-localization [18].

In this study, 1133 lncRNAs were identified for the first time on a genome-wide scale, using a set of published next-generation RNA-*seq* data from five tissues of mulberry. Furthermore, the structural characteristics and tissue specificity of the predicted lncRNAs were analyzed and compared with the mRNAs. Additionally, the functions of the novel lncRNAs were predicted based on genomic positioning information, which was important for further clarifying the roles of the lncRNAs in the growth and development of woody plants.

## 2. Experimental Section

### 2.1. The Pipeline to Identify lncRNAs from RNA-seq Data

**Figure 1:**
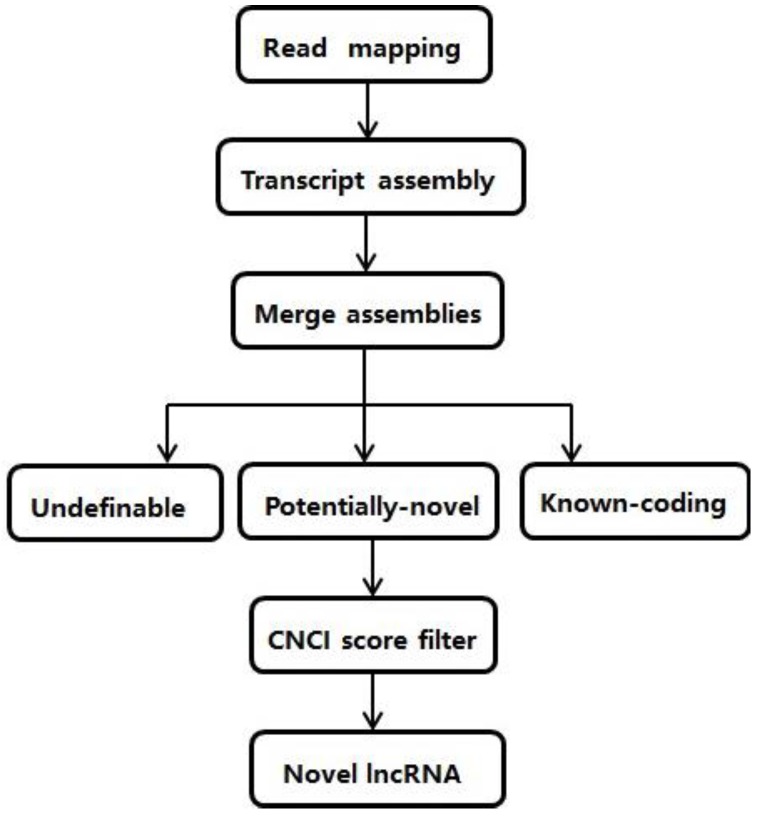
Pipeline to identify lncRNAs from RNA-*seq* data.

A set of *Morus notabilis* clean RNA-*seq* data with a length of 90bp and taken from five different tissues was obtained from a published study [28] and downloaded from the NCBI SRA website with the project number SRX504906. The protein-coding genes of RefSeq [29], Ensembl [30], UCSC [31], and Vega [32] were downloaded from the UCSC genome browser and all known noncoding genes from the NONCODE4.0 database [33]. The mulberry reference genome and gene model annotation files were downloaded from the genome website [28], and a pipeline was developed to identify putative lncRNAs (Figure 1).

After filtering out low-quality reads, the spliced read aligner TopHat version 2.0.9 [34] was used to map all clean reads to the mulberry genome. We used two rounds of TopHat mapping to maximize the usage of the splice junction information from all RNA-*seq* data. In the first round, all reads were mapped with TopHat (parameters: min-anchor = 5, min-isoform-fraction = 0, and other parameters with default values); in the second round of TopHat remapping, all splice junctions produced by the initial mapping were fed into TopHat to map reads (parameters: raw-juncs, no-novel-juncs, and min-anchor = 5, and min-isoform-fraction = 0).

Mapped reads from TopHat for each tissue were assembled for each sample separately by Cufflinks [35]. The cufflinks employed spliced read information to determine exon connectivity. Specifically, it used a probabilistic model approach to assemble and quantify the expression level of a minimal set of isoforms and provided the maximum level of annotation on the expression data for given loci. Cufflinks version 2.1.1 was run with default parameters (except “min-frags-per-transfrag = 0”). The multiple assembled transcript files for different tissues were then merged together to produce a unique transcriptome set using Cuffmerge.

We then used an analysis process to minimize false positives and maximize the number of lncRNAs from the merged transcripts, which included the following steps: (1) compare the merged transcripts with known protein-coding genes and lncRNAs in the public databases; (2) select transcripts that are longer than 200 nt; and (3) filter the putative lncRNA transcripts by coding potential using CNCI software [36], which can be categorized as noncoding (CNCI is a powerful signature tool that profiles adjoining nucleotide triplets to effectively distinguish protein-coding and non-coding sequences independent of known annotations) [37].

### 2.2. Calculation of lncRNA Conservation

To further demonstrate the reliability of lncRNAs predicted from the RNA-*seq* data and calculate the conservation of the novel lncRNAs, a set of lncRNAs collected by TAIR [38] and PlncDB [39] was downloaded and then aligned with the sequences of novel mulberry lncRNAs using BLASTN software [40].

### 2.3. Expression Profiles of Tissue Specific lncRNAs and Functional Predictions

To evaluate the tissue specificity of a transcript, we devised an entropy-based method to quantify the similarity between a transcript’s expression pattern and another predefined pattern, which represented an extreme case where a transcript was expressed in only one tissue [41]. After obtaining the lncRNA dataset with tissue-specific expression, we further searched the genomic location information from the genome comparison results by running a script with Perl, and retrieved the information of coding genes within the scope of its ±10 Kb.

### 2.4. qRT-PCR Analysis of lncRNAs

Three individuals of mulberry were used as biological replicates. Tissues from bark, root and winter bud were isolated with a sharp chisel and frozen immediately in liquid nitrogen. Total RNA was extracted with Universal Total RNA Kit (BioTeke, Beijing, China). First-strand cDNA synthesis was carried out with approximately 1.0 μg RNA using the Prime Script™ RT Master Mix (Takara, Dalian, China). All primers used in this study are listed in Supplementary Materials Table S1. Real-time qRT-PCR was performed in quadruplicate using the SYBR Premix Ex Taq™ II Kit (Takara, Dalian, China) on a Roche light Cycler 480 (Roche Applied Science, Penzberg, Upper Bavaria, Germany) according to the manufacturer’s instructions. Sample cycle threshold (Ct) values were determined and standardized relative to the endogenous control genes ACTIN3, and the 2–∆∆CT method was used to calculate the relative changes in gene expression based on the qRT-PCR data [42].

## 3. Results

### 3.1. Transcripts Reconstruction and Identification of Novel lncRNAs

The RNA-*seq* data used in this study were downloaded from the NCBI SRA website. These reads were paired and both lengths were 90 nt. Starting from a total of 1.2 billion reads, we performed short read gapped alignment using TopHat [34] and recovered 1.01 billion (84%) mapped reads (Table 1).

We then used Cufflinks [35] to *de novo* reconstruct one set of transcripts for each tissue based on the read-mapping results. Transcripts reconstructed were separately merged into combined sets of transcripts using the Cuffcompare utility provided by Cufflinks. After filtering for exon number, transcript length, and coverage, we obtained 41,042 reliably expressed transcripts (Table 2).

**Figure 2:**
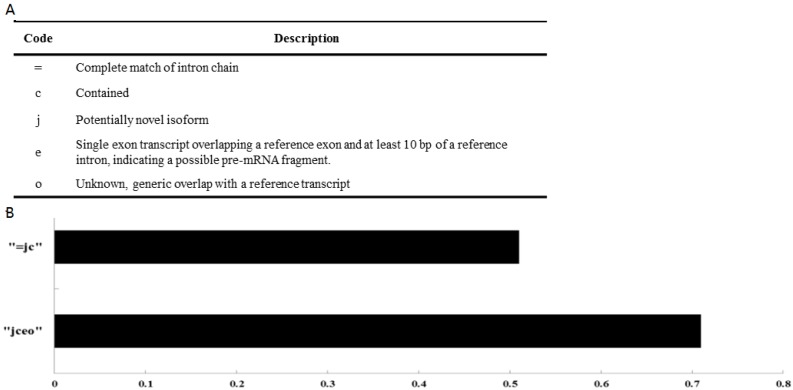
(**A**) Different classes of assembled transcripts according to their relative positions with known coding genes; (**B**) Ratio of the reconstructed transcripts to known coding genes.

To assess the robustness of these *ab initio* assemblers, we analyzed their performance on protein-coding genes. The transcripts we reconstructed using Cufflinks covered 70.79% of known mulberry coding genes (Figure 2). These results strongly supported the fact that these assembly approaches could robustly and reliably reconstruct both coding and noncoding transcripts at a global level.

**Figure 3:**
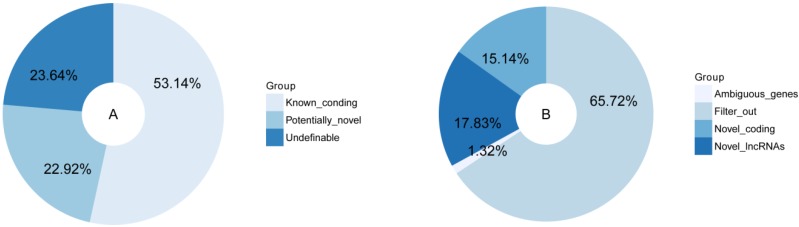
Classification of reconstructed transcripts (**A**) and results from CNCI (**B**).

Based on the robust transcript reconstruction and broad availability of deep sequencing datasets, we used an analysis process to minimize the false positives and maximize the number of lncRNA transcripts, compared the merged transcripts with known protein-coding genes and lncRNAs in the public databases, and classified the combined transcripts into several different subsets. The majority of the transcripts (53.44%) corresponding to the annotated protein-coding genes, while the rest of the transcripts were undefinable (23.64%), and potentially novel (22.92%). The potentially novel transcripts were then filtered for coding potential based on CNCI software [43], resulting in the identification of 1133 reliably expressed lncRNAs with length >200 nt (Figure 3).

**Figure 4:**
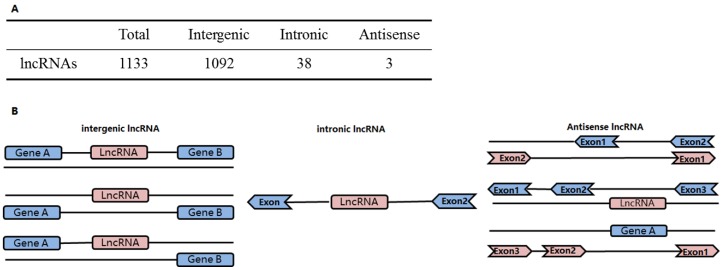
Classification of the predicted lncRNAs. (**A**) lncRNAs were classified as intergenic, intronic or antisense lncRNAs based on the spatial relationships of their gene loci with protein-coding genes; (**B**) Schematic illustration of the classification of lncRNA genes based on their spatial relationship with protein-coding genes.

The identified lncRNAs were classified as intergenic, intronic and antisense lncRNAs based on spatial relationships of their gene loci with protein-coding genes (Figure 4B). The identified lncRNAs were mostly intergenic lncRNAs, with 1092 in total, accounting for 96.4% of the identified lncRNA. There were 38 intronic lncRNAs, accounting for 3.4%, and 3 antisense lncRNAs, accounting for 0.26% (Figure 4A).

### 3.2. Characterization of the Novel lncRNAs

**Figure 5:**
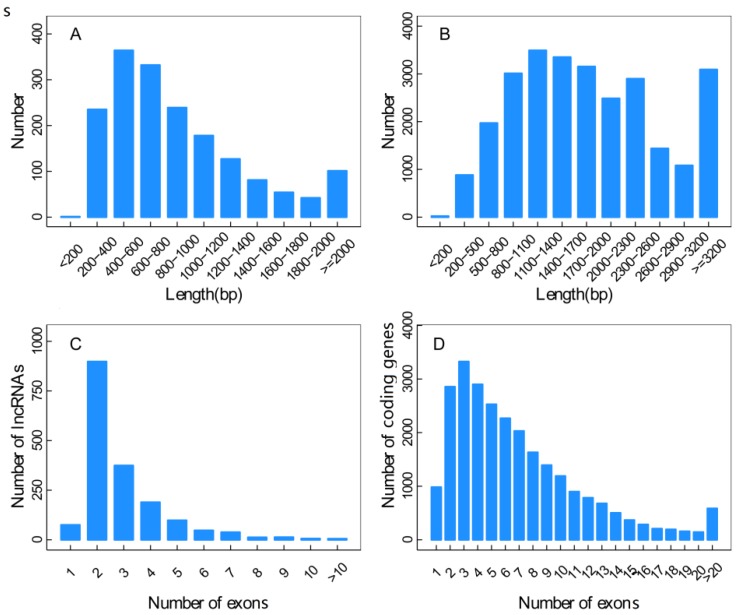
Sequence features of lncRNAs. (**A**) and (**B**) represented the length distributions of the transcripts from novel lncRNAs (**A**) and known coding genes (**B**); (C) and (D) represented the exon numbers of the transcripts of lncRNAs (**C**) and known coding genes (**D**).

The length distribution results showed that the novel identified 1133 lncRNAs contained 1755 transcripts mainly in the range of 200–1200 bp. The lengths of 25,902 transcripts from known coding genes were greater than that of the lncRNAs, mostly above 800 bp. The distribution results of exon numbers revealed that there were 982 single exons (3.79%) and 24,920 multi-exons (96.21%) in the 25,902 transcripts from known coding genes. There were 75 single exons (4.27%) and 1680 multi-exons (95.73%) in the lncRNA 1755 transcripts, revealing a similar proportion of multi-exons to the known coding genes (Figure 5).

**Figure 6:**
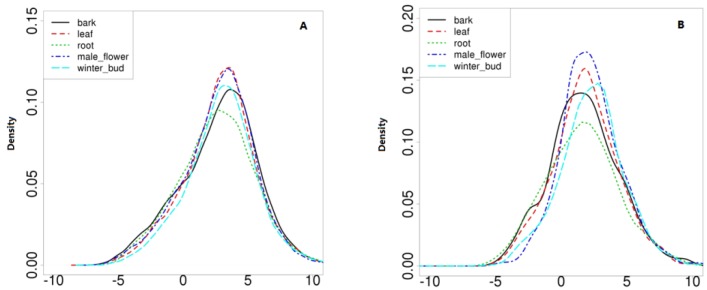
Comparison of expression levels between known coding genes and lncRNAs. (**A**) represents the density of expression of known-coding genes *N* = 26965, Bandwidth = 0.3673; and (**B**) represents the lncRNAs, *N* = 1005, Bandwidth = 0.5891.

In combination with all known lncRNAs, we established a comprehensive catalog of 1133 transcribed lncRNA genes. Based on the Fragments Per Kilobase of transcript per Million mapped reads (FPKM) of each transcript, calculated by “Cufflinks” “abundance estimation mode” across the five tissues, we compared the expression differences between lncRNAs and protein-coding genes. The average expression levels of lncRNAs were lower than those for protein coding genes, but lncRNAs showed a wider range of abundance, with a subset of them equally abundant to mRNAs (Figure 6).

**Figure 7:**
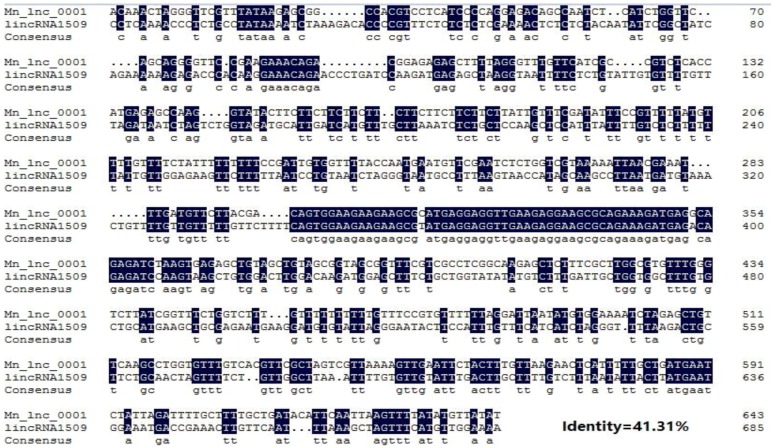
Alignment of the nucleotide sequences of Mn_lnc_0001 and lincRNA1509. Black and white backgrounds indicate conserved and non-conserved residues, respectively.

Through conservation analysis we found that 112 lncRNAs from the 1133 newly identified genes had homologies in the Arabidopsis database, while only 9 lncRNAs had homologies in the poplar database (Supplementary Table S2). The homology comparison results of the novel lncRNAs of mulberry with the mapped poplar lncRNAs confirmed the high level of homology between two sequences as 41.31% (Figure 7).

### 3.3. Expression Profiles of Tissue Specific lncRNAs and Functional Predictions

**Figure 8:**
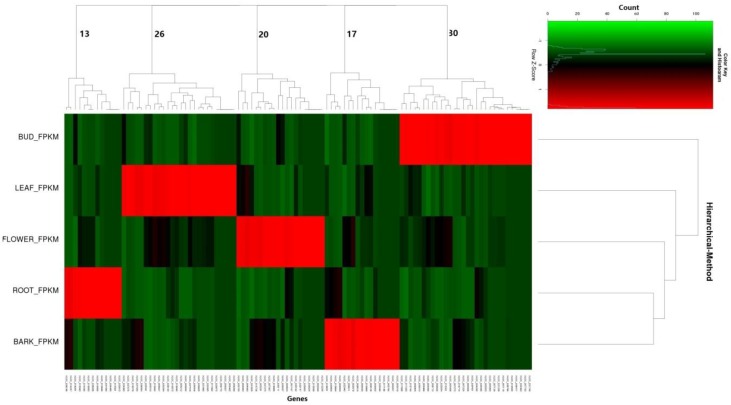
Tissue specificity of lncRNAs from winter bud, leaf, male flower, root, and bark of mulberry.

To assess the tissue specificity of mulberry lncRNA expression, we calculated the Jensen-Shannon tissue specificity score (JS score) [40] for each transcript using the established procedure. Using a JS score = 0.9 as a cutoff, we demonstrated that only 9.35% of the lncRNAs were tissue-specific (Figure 8). Thus, some of the lncRNA expressions of mulberry were clearly subject to tissue dependent regulation, either at the level of transcription or degradation.

Comparing their genomic locations with those of known mulberry coding genes, we found that among the 1133 lncRNAs, 106 (9.35%) were tissue-specific, including 82 lncRNAs adjacent (±10 kb) to protein-coding genes (Supplementary Materials Table S3–S7). The functions annotated to the protein-coding genes mainly involved hormone signal recognition and transduction, plant secondary metabolite synthesis, energy metabolism, *etc.* The lncRNAs Mn_lnc_0132, Mn_lnc_0521, and Mn_lnc_0782 were specifically expressed in male flower: Mn_lnc_0132 was located near the protein-coding gene EXB28594.1 (Protein PROLIFERA). Protein PROLIFERA, a highly conserved protein, was found in all eukaryotes, and specifically expressed in populations of dividing cells in sporophytic tissues of the plant body, such as the palisade layer of the leaf and founder cells of initiating flower primordia [44]. Mn_lnc_0521 was located near EXB81017.1 (Serine/threonine-protein phosphatase PP). The PP1s were shown to play key roles in many aspects of plant growth and development, such as pollination and pollen tube development [45,46,47]. It was found that Mn_lnc_0782 was located near EXC20310.1 (Phosphoenolpyruvate/phosphate translocator PPT). Located in the plastid, PPT played a pivotal role in the regulation of leaf color, florescence, and female and male gametophyte formation [48,49,50,51]. A sulfate transporter, Mn_lnc_0714, was specifically expressed in root and located near EXC06697.1 (Sulfate transporter 1.3). It was tissue-specifically expressed and was crucial for root development and symbiotic nitrogen fixation in root nodules [52,53,54].

**Figure 9:**
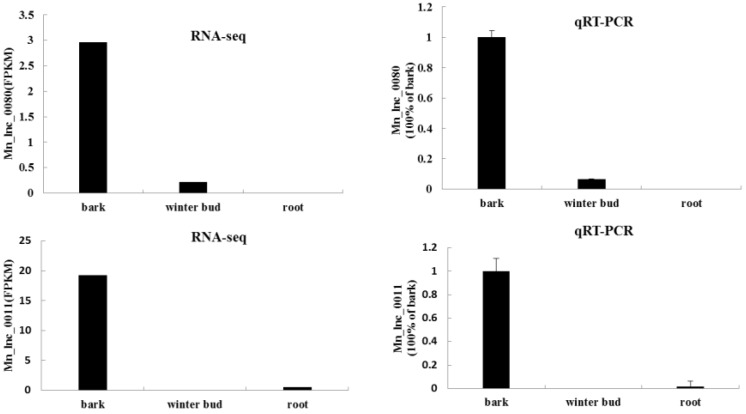
Typical result of qRT-PCR verification. Transcripts abundance based on RNA-*seq* (left) and qRT-PCR (right) is shown for lncRNAs identified form RNA-*seq* data.

To validate RNA-*seq* results, qRT-PCR were performed for 10 randomly selected tissue-specific lncRNAs in bark. As a result, all 10 reactions generated sequence products. Remarkable higher relative quantitative expressions of the 10 lncRNA were observed in bark. However, only 2 and 4 of the 10 lncRNAs expressed in winter bud and root, respectively, but their expression levels were quite low, ranging from 1.4% to 15.4% of the expression level in bark (Figure 9).

## 4. Discussion

An avalanche of RNA-*seq* data emerged as powerful high-throughput sequencing technologies became more pervasive and user-friendly. However, systematic identification of lncRNAs was limited to only a few plant species [21,26,27,55,56], leaving most plant transcriptome sequencing data not fully explored, even though these novel molecules play important roles in a wide range of biological processes [15]. Because lncRNAs are generated by the same transcriptional machinery as mRNAs [57], no defining biochemical features could be exclusively ascribed to lncRNAs, such as a 5′ cap, 3′ polyadenylated tail, and splicing [58]. Defining lncRNAs simply on the basis of size and lack of protein-coding capability was intellectually far from satisfying. In this paper, we designed a strict computational pipeline and identified 1133 novel lncRNAs from the entire genome using a set of published mulberry next-generation RNA-*seq* data. The pipeline used in this study can be easily adapted to other organisms, especially for species that have not been well studied to date.

The expression levels of the novel mulberry lncRNAs in root, leaf, bark, bud, and male flower were below the expression levels of mRNAs, which was consistent with findings in other species [59,60,61]. Conservation analysis found that among the 1133 lncRNAs, 112 (9.4%) had homology in the Arabidopsis database, and 9 (0.8%) had homology in the poplar lncRNA database. The low levels of conservation might be caused by the incomplete lncRNA databases of plants. The results also reflected the less restrictive factors on the evolution of lncRNAs, and thus the low conservation levels of lncRNA sequences among species, factors that reduce the possibility of forming a large family with homologous genes. Moreover, qRT-PCR was performed, and the RNA-*seq* results were consistent with the qRT-PCR data, providing further proof that the prediction accuracy was sufficient.

Numerous studies have shown that lncRNAs with tissue-specific expression usually had special functions [62], and the lncRNAs of higher species primarily played the biological role of *cis*-regulation of the adjacent genes [63,64,65]. In the analysis of tissue-specific expression, we found that 106 lncRNAs from our 1133 newly identified genes were expressed specifically in five separate tissues, among which 82 had known protein-coding genes in the range of ±10 Kb. We therefore predicted the functions of these lncRNAs by analysis of the tissue-specific expressions and the functions of adjacent coding genes. Further analysis showed that three male flower-specific lncRNAs were located adjacent to coding genes, which are related to development of floral organs. One root-specific lncRNA was located adjacent to a coding gene, which is crucial for root development and symbiotic nitrogen fixation. These results suggest that these novel lncRNAs might play important regulatory roles in the development of floral organs and root in mulberry.

Regarding the important functions of lncRNAs in plant growth and development, their identification within plant-wide genomes is rapidly developing. By contrast, the functional characterization of lncRNAs for plants is far behind that of other species. So far, the commonly used methods for lncRNA functional prediction are based on co-expression networks [57], miRNA regulation [66], protein binding [67], epigenetic modification [68], and adjacent gene functions. In this study, due to the influence of sequencing data (insufficient sample size) we cannot make functional predictions through the methods of co-expression networks and others. These methods are only based on the functional predictions of bioinformatics, so the accurate assignment of functions of lncRNAs still requires verification through biological experiments. However, with the development of biotechnology and more information becoming known about lncRNAs, their important functions in plant growth and development will be uncovered gradually.

## Figures and Tables

**Table 1:** RNA-*seq* data production and alignment results for reads of different tissues.

Sample	Total Reads	Left Mapped Reads		Right Mapped Reads		Total Mapped Reads	
Bark	25,992,683	22,547,116	86.74%	22,221,501	85.49%	23,847,766	91.75%
Leaf	24,809,215	22,686,967	91.45%	22,244,419	89.66%	24,123,695	97.24%
Root	21,483,404	16,972,204	79.00%	16,637,319	77.44%	18,039,734	83.97%
Male flower	26,629,083	24,015,382	90.18%	23,545,895	88.42%	25,681,360	96.44%
Winter bud	18,138,525	14,706,155	81.08%	14,392,578	79.35%	15,841,259	87.33%

**Table 2:** Exon numbers of reconstructed transcripts.

Sample	Junctions	Transcripts	Multi Exon	Multi Exon/Transcripts
Bark	108814	30009	21907	73.00%
Leaf	105808	32664	23354	71.50%
Root	86084	28632	20163	70.42%
Male flower	108894	35616	24368	68.42%
Winter bud	75878	35553	21654	60.91%
Merge		41042	30429	74.14%

## Supplementary Materials

The following are available online at http://www.mdpi.com/2073-4425/7/3/11/s1. Supplementary Table S1–S7.
