# Supplementary material for: Genome-Wide Identification and Characterization of Long Non-Coding RNAs from Mulberry (Morus notabilis) RNA-seq Data
Source: Genes (Basel). 2016 Feb 29;7(3):11. doi: 10.3390/genes7030011 (PMC4808792; doi:10.3390/genes7030011)
Supplement: Supplementary file 1 [file genes-07-00011-s001.docx]

Supplementary Materials: Genome-Wide Identification and Characterization of Long
Non-Coding RNAs from Mulberry
(*Morus notabilis*) RNA-*seq* Data

Xiaobo Song Liang Sun, Haitao Luo, Qingguo Ma, Yi Zhao and Dong Pei

**Table S1.** Primers used in this study.

| **NO** | **Name** | **Sequence(5′-3′)** |
| --- | --- | --- |
| **1** | **MnACTIN3-F** | GCATGAAGATCAAGGTGGTG |
| **2** | **MnACTIN3-R** | CATCTGCTGGAAGGTGCTAA |
| **3** | **XLOC_000003F** | CCATGTGAGATCATCCACC |
| **4** | **XLOC_000003R** | ACTTGTGTTTAGTTCTGGGAAT |
| **5** | **XLOC_022903F** | CTAGAAGTTGAGATTTGCCG |
| **6** | **XLOC_022903R** | GAGAGAAGGAGGGAAACCA |
| **7** | **XLOC_019631F** | TGCTGAGACAACATTAGACG |
| **8** | **XLOC_019631R** | AATAACTCTCTCGCCACC |
| **9** | **XLOC_003002F** | GAGGAACGGGCTAAGATG |
| **10** | **XLOC_003002R** | ATTTCACTGATTCTGATTGCG |
| **11** | **XLOC_005792F** | AGCAACATTGATCTGTTCATAG |
| **12** | **XLOC_005792R** | CCAAGAACCGACTTCACC |
| **13** | **XLOC_000207F** | CATCTTCTAATCTCCATGCACT |
| **14** | **XLOC_000207R** | TTTATCCTTAGGCAGGCTTG |
| **15** | **XLOC_017186F** | TCACACAAATCCAAGATGGAAC |
| **16** | **XLOC_017186R** | CTTTGTCTGTCCTCTTAACTGA |
| **17** | **XLOC_009108F** | TGATCTGCACCAACCATTC |
| **18** | **XLOC_009108R** | ATAGCTGCAACAAGAGCGA |
| **19** | **XLOC_022049F** | TATGCACATAAGACTTAGCAGC |
| **20** | **XLOC_022049R** | TCAAGTTGTAGAAAGCACGG |
| **21** | **XLOC_003519F** | GAGCTAAGCCAACCTAGTAAC |
| **22** | **XLOC_003519R** | GAGAATCTTACCTGTCCGTG |
| **23** | **XLOC_013694F** | GTTAGTCGACGACCCATTC |
| **24** | **XLOC_013694R** | ATTTAGTTGACGCGGGTT |
| **25** | **XLOC_009918F** | GCTCACTAGCAAGGGAATGATA |
| **26** | **XLOC_009918R** | CTCACAAGAAGCCGGTAA |
| **27** | **XLOC_009911F** | TGAGGAATAACTGCTGGGAAA |
| **28** | **XLOC_009911R** | GACCCACACGTAGAATAATACT |
| **29** | **XLOC_003878F** | TCTGTATTCTTGTATGGAGGCA |
| **30** | **XLOC_003878R** | AAGGGACTTCAACTCAATGAAA |
| **31** | **XLOC_010969F** | ATTTGTCCAAGAGCAGGCA |
| **32** | **XLOC_010969R** | TCTCCACTGTTCTCGTCG |
| **33** | **XLOC_022840F** | TGTGAAGTCAGTCAGCTCC |
| **34** | **XLOC_022840R** | GACTAAGGTTGTGGGTCTTATT |
| **35** | **XLOC_000033F** | AGAACCTAAACCTGCTGG |
| **36** | **XLOC_000033R** | CCTTGTGCTTTGGAACTAC |

**Table S2.** Result of conservation analysis.

| **LncRNA ID** | **Mapped P.tri LncRNA NO** | **Mapped P.tri LncRNA List** | **Mapped Tair LncRNA NO** | **Mapped Tair LncRNA List** |
| --- | --- | --- | --- | --- |
| Mn_lnc-0001 | 3 | lincRNA1509  lincRNA2088  lincRNA2100 | 1 | NONATHT000414 |
| Mn_lnc-0002 | 1 | lincRNA1270 | 66 | NONATHT000064,NONATHT000068…… |
| Mn_lnc-0003 | 1 | lincRNA2062 | 7 | NONATHT000970,NONATHT001685…… |
| Mn_lnc-0004 | 1 | lincRNA2062 | 7 | NONATHT000970,NONATHT001685…… |
| Mn_lnc-0005 | 1 | lincRNA3306 | 5 | NONATHT000140,NONATHT003062…… |
| Mn_lnc-0006 | 1 | lincRNA1209 | 3 | NONATHT000984,NONATHT001209…… |
| Mn_lnc-0010 | 0 | — | 27 | NONATHT000043,NONATHT000052…… |
| Mn_lnc-0011 | 0 | — | 23 | NONATHT000028,NONATHT000045…… |
| Mn_lnc-0012 | 0 | — | 8 | NONATHT000436,NONATHT000970, …… |
| Mn_lnc-0013 | 0 | — | 8 | NONATHT000436,NONATHT000970…… |
| Mn_lnc-0014 | 0 | — | 7 | NONATHT000815,NONATHT000984…… |
| Mn_lnc-0015 | 0 | — | 6 | NONATHT000984,NONATHT001685…… |
| Mn_lnc-0016 | 0 | — | 5 | NONATHT000414,NONATHT000815…… |
| Mn_lnc-0017 | 0 | — | 5 | NONATHT000436,NONATHT000984…… |
| Mn_lnc-0018 | 0 | — | 4 | NONATHT002060,NONATHT003599…… |
| Mn_lnc-0019 | 0 | — | 3 | NONATHT001309,NONATHT002122…… |
| Mn_lnc-0020 | 0 | — | 3 | NONATHT000970,NONATHT000984, …… |
| Mn_lnc-0021 | 0 | — | 3 | NONATHT000436,NONATHT001311…… |
| Mn_lnc-0022 | 0 | — | 3 | NONATHT000436,NONATHT001311…… |
| Mn_lnc-0023 | 0 | — | 3 | NONATHT000984,NONATHT001685…… |
| Mn_lnc-0024 | 0 | — | 3 | NONATHT000984,NONATHT001685…… |
| Mn_lnc-0025 | 0 | — | 3 | NONATHT000984,NONATHT002122…… |
| Mn_lnc-0026 | 0 | — | 3 | NONATHT000970,NONATHT001309…… |
| Mn_lnc-0027 | 0 | — | 3 | NONATHT001730,NONATHT002060…… |
| Mn_lnc-0028 | 0 | — | 3 | NONATHT000970,NONATHT000984…… |
| Mn_lnc-0029 | 0 | — | 2 | NONATHT001730,NONATHT002010 |
| Mn_lnc-0030 | 0 | — | 2 | NONATHT000436,NONATHT003487 |
| Mn_lnc-0031 | 0 | — | 2 | NONATHT000984,NONATHT002122 |
| Mn_lnc-0032 | 0 | — | 2 | NONATHT000970,NONATHT003487 |
| Mn_lnc-0033 | 0 | — | 2 | NONATHT002060,NONATHT003295 |
| Mn_lnc-0034 | 0 | — | 2 | NONATHT002060,NONATHT003295 |
| Mn_lnc-0035 | 0 | — | 2 | NONATHT001730,NONATHT002060 |
| Mn_lnc-0036 | 0 | — | 2 | NONATHT000970,NONATHT001690 |
| Mn_lnc-0037 | 0 | — | 2 | NONATHT001685,NONATHT002060 |
| Mn_lnc-0038 | 0 | — | 2 | NONATHT001685,NONATHT002060 |
| Mn_lnc-0039 | 0 | — | 2 | NONATHT002060,NONATHT003295 |
| Mn_lnc-0040 | 0 | — | 2 | NONATHT003541,NONATHT003542 |
| Mn_lnc-0041 | 0 | — | 2 | NONATHT000815,NONATHT001689 |
| Mn_lnc-0042 | 0 | — | 2 | NONATHT001685,NONATHT002010 |
| Mn_lnc-0043 | 0 | — | 2 | NONATHT000414,NONATHT001309 |
| Mn_lnc-0044 | 0 | — | 2 | NONATHT000414,NONATHT001690 |
| Mn_lnc-0045 | 0 | — | 2 | NONATHT002122,NONATHT003487 |
| Mn_lnc-0046 | 0 | — | 2 | NONATHT002392,NONATHT003576 |
| Mn_lnc-0047 | 0 | — | 2 | NONATHT003836,NONATHT003850 |
| Mn_lnc-0048 | 0 | — | 2 | NONATHT000414,NONATHT003610 |
| Mn_lnc-0049 | 0 | — | 2 | NONATHT001617,NONATHT003295 |
| Mn_lnc-0050 | 0 | — | 2 | NONATHT001685,NONATHT003487 |
| Mn_lnc-0051 | 0 | — | 2 | NONATHT003541,NONATHT003542 |
| Mn_lnc-0052 | 0 | — | 2 | NONATHT003839,NONATHT003847 |
| Mn_lnc-0053 | 0 | — | 2 | NONATHT000970,NONATHT000984 |
| Mn_lnc-0054 | 0 | — | 2 | NONATHT000436,NONATHT002122 |
| Mn_lnc-0055 | 0 | — | 2 | NONATHT003045,NONATHT003046 |
| Mn_lnc-0056 | 0 | — | 2 | NONATHT002060,NONATHT003295 |
| Mn_lnc-0057 | 0 | — | 1 | NONATHT003295 |
| Mn_lnc-0058 | 0 | — | 1 | NONATHT003144 |
| Mn_lnc-0059 | 0 | — | 1 | NONATHT001311 |
| Mn_lnc-0060 | 0 | — | 1 | NONATHT001730 |
| Mn_lnc-0061 | 0 | — | 1 | NONATHT002060 |
| Mn_lnc-0062 | 0 | — | 1 | NONATHT002122 |
| Mn_lnc-0063 | 0 | — | 1 | NONATHT003295 |
| Mn_lnc-0064 | 0 | — | 1 | NONATHT002779 |
| Mn_lnc-0065 | 0 | — | 1 | NONATHT000436 |
| Mn_lnc-0066 | 0 | — | 1 | NONATHT003487 |
| Mn_lnc-0067 | 0 | — | 1 | NONATHT003295 |
| Mn_lnc-0068 | 0 | — | 1 | NONATHT001730 |
| Mn_lnc-0069 | 0 | — | 1 | NONATHT001721 |
| Mn_lnc-0070 | 0 | — | 1 | NONATHT001721 |
| Mn_lnc-0071 | 0 | — | 1 | NONATHT003610 |
| Mn_lnc-0072 | 0 | — | 1 | NONATHT001311 |
| Mn_lnc-0073 | 0 | — | 1 | NONATHT003295 |
| Mn_lnc-0074 | 0 | — | 1 | NONATHT001824 |
| Mn_lnc-0075 | 0 | — | 1 | NONATHT003487 |
| Mn_lnc-0076 | 0 | — | 1 | NONATHT001311 |
| Mn_lnc-0077 | 0 | — | 1 | NONATHT001685 |
| Mn_lnc-0078 | 0 | — | 1 | NONATHT001082 |
| Mn_lnc-0079 | 0 | — | 1 | NONATHT003295 |
| Mn_lnc-0080 | 0 | — | 1 | NONATHT000984 |
| Mn_lnc-0081 | 0 | — | 1 | NONATHT003295 |
| Mn_lnc-0082 | 0 | — | 1 | NONATHT000984 |
| Mn_lnc-0083 | 0 | — | 1 | NONATHT002060 |
| Mn_lnc-0084 | 0 | — | 1 | NONATHT000970 |
| Mn_lnc-0085 | 0 | — | 1 | NONATHT001721 |
| Mn_lnc-0086 | 0 | — | 1 | NONATHT002060 |
| Mn_lnc-0087 | 0 | — | 1 | NONATHT001311 |
| Mn_lnc-0088 | 0 | — | 1 | NONATHT001311 |
| Mn_lnc-0089 | 0 | — | 1 | NONATHT001311 |
| Mn_lnc-0090 | 0 | — | 1 | NONATHT001311 |
| Mn_lnc-0091 | 0 | — | 1 | NONATHT003295 |
| Mn_lnc-0092 | 0 | — | 1 | NONATHT001311 |
| Mn_lnc-0093 | 0 | — | 1 | NONATHT001309 |
| Mn_lnc-0094 | 0 | — | 1 | NONATHT003732 |
| Mn_lnc-0095 | 0 | — | 1 | NONATHT001418 |
| Mn_lnc-0096 | 0 | — | 1 | NONATHT001309 |
| Mn_lnc-0097 | 0 | — | 1 | NONATHT000984 |
| Mn_lnc-0098 | 0 | — | 1 | NONATHT000984 |
| Mn_lnc-0099 | 0 | — | 1 | NONATHT000984 |
| Mn_lnc-0100 | 0 | — | 1 | NONATHT003295 |
| Mn_lnc-0101 | 0 | — | 1 | NONATHT001309 |
| Mn_lnc-0102 | 0 | — | 1 | NONATHT000984 |
| Mn_lnc-0103 | 0 | — | 1 | NONATHT001800 |
| Mn_lnc-0104 | 0 | — | 1 | NONATHT000984 |
| Mn_lnc-0105 | 0 | — | 1 | NONATHT002073 |
| Mn_lnc-0106 | 0 | — | 1 | NONATHT000984 |
| Mn_lnc-0107 | 0 | — | 1 | NONATHT000984 |
| Mn_lnc-0108 | 0 | — | 1 | NONATHT003295 |
| Mn_lnc-0109 | 0 | — | 1 | NONATHT001721 |
| Mn_lnc-0110 | 0 | — | 1 | NONATHT002122 |
| Mn_lnc-0111 | 0 | — | 1 | NONATHT003295 |
| Mn_lnc-0112 | 0 | — | 1 | NONATHT002122 |
| Mn_lnc-0113 | 0 | — | 1 | NONATHT002060 |
| Mn_lnc-0114 | 0 | — | 1 | NONATHT000730 |
| Mn_lnc-0115 | 0 | — | 1 | NONATHT003295 |

(XBS)

**Table S3.** Summary of tissue specific genes in root with neighbor coding genes and functions.

| **LncRNA ID** | **Neighbor Coding Gene** | **Function** |
| --- | --- | --- |
| Mn_lnc-0041 | EXB66525.1,  EXB66527.1,  EXB66528.1 | Phosphatidylinositol-glycan biosynthesis class X protein Mitochondrial inner membrane protease ATP23，hypothetical protein Mitogen-activated protein kinase kinase 5 |
| Mn_lnc-0042 | EXB72255.1 | hypothetical protein |
| Mn_lnc-0050 | EXB87346.1 | putative amino acid permease YfnA |
| Mn_lnc-0053 | EXB93293.1 | hypothetical protein |
| Mn_lnc-0056 | EXB94111.1 | putative NADH dehydrogenase |
| Mn_lnc-0057 | EXB95198.1 | hypothetical protein |
| Mn_lnc-0059 | EXB97271.1 | Non-specific lipid-transfer protein-like protein |
| Mn_lnc-0065 | EXC06697.1 | Sulfate transporter 1.3 |
| Mn_lnc-0087 | EXC30709.1 | Werner Syndrome-like exonuclease |
| Mn_lnc-0091 | EXC31783.1 | 4-coumarate--CoA ligase-like 7 |

(XBS)

**Table S4.** Summary of tissue specific genes in bark with neighbor coding genes and functions.

| **LncRNA ID** | **Neighbor Coding Gene** | **Function** |
| --- | --- | --- |
| Mn_lnc-0001 | EXB19758.1 | Ethylene-responsive transcription factor |
| Mn_lnc-0002 | EXB20730.1 | hypothetical protein |
| Mn_lnc-0003 | EXB23830.1 | Auxin-binding protein ABP19a |
| Mn_lnc-0011 | EXB38292.1,  EXB38293.1 | 60S ribosomal protein L6 Phosphatidylinositol-4-phosphate 5-kinase 4 |
| Mn_lnc-0014 | EXB39149.1 | hypothetical protein |
| Mn_lnc-0015 | EXB40022.1 | hypothetical protein |
| Mn_lnc-0024 | EXB51228.1 | putative LRR receptor-like serine/ threonine-protein kinase |
| Mn_lnc-0037 | EXB62622.1 | UDP-glycosyltransferase 85A |
| Mn_lnc-0039 | EXB65570.1 | hypothetical protein |
| Mn_lnc-0043 | EXB72448.1 | Curved DNA-binding protein |
| Mn_lnc-0049 | EXB82808.1 | Polyphenol oxidase |
| Mn_lnc-0066 | EXC10952.1 | DNA polymerase III subunit |
| Mn_lnc-0075 | EXC21500.1 | hypothetical protein |
| Mn_lnc-0080 | EXC25147.1 | hypothetical protein |
| Mn_lnc-0082 | EXC25309.1, EXC25310.1 | Isoleucine N-monooxygenase 2 Type II inositol-1%2C4%2C5-trisphosphate 5-phosphatase FRA3 |

(XBS)

**Table S5.** Summary of tissue specific genes in winter bud with neighbor coding genes and functions.

| **LncRNA ID** | **Neighbor Coding Gene** | **Function** |
| --- | --- | --- |
| Mn_lnc-0004 | EXB26546.1 | hypothetical protein |
| Mn_lnc-0005 | EXB27067.1,  EXB27068.1,  EXB27070.1 | putative glutathione S-transferase parC  putative glutathione S-transferase  putative glutathione S-transferase |
| Mn_lnc-0009 | EXB36838.1,  EXB36842.1 | Dihydrolipoyllysine-residue acetyltransferase component 2 of pyruvate dehydrogenase complex  ER lumen protein retaining receptor |
| Mn_lnc-0012 | EXB38871.1 | Casein kinase I isoform delta-like protein |
| Mn_lnc-0017 | EXB41586.1 | F-box protein |
| Mn_lnc-0025 | EXB51430.1 | hypothetical protein |
| Mn_lnc-0028 | EXB53993.1 | GC-rich sequence DNA-binding factor 1 |
| Mn_lnc-0030 | EXB54658.1 | hypothetical protein |
| Mn_lnc-0031 | EXB54658.1 | hypothetical protein |
| Mn_lnc-0032 | EXB54869.1 | hypothetical protein |
| Mn_lnc-0033 | EXB54966.1 | hypothetical protein |
| Mn_lnc-0034 | EXB56221.1 | NAD(P)H-quinone oxidoreductase subunit 3 |
| Mn_lnc-0035 | EXB56922.1 | hypothetical protein |
| Mn_lnc-0051 | EXB88542.1 | Glutamate receptor 2.7 |
| Mn_lnc-0052 | EXB93127.1, EXB93128.1 | GDSL esterase/lipase,  G-type lectin S-receptor-like serine/ threonine-protein kinase |
| Mn_lnc-0063 | EXC01148.1 | MATE efflux family protein 5 |
| Mn_lnc-0067 | EXC11337.1 | Wall-associated receptor kinase-like 20 |
| Mn_lnc-0070 | EXC16242.1 | Ubiquitin-40S ribosomal protein S27a |
| Mn_lnc-0071 | EXC19487.1,  EXC19488.1 | 40S ribosomal protein S23  RNA-binding protein 38 |
| Mn_lnc-0073 | EXC20544.1 | MATE efflux family protein DTX1 |
| Mn_lnc-0078 | EXC24413.1 | hypothetical protein |
| Mn_lnc-0085 | EXC28022.1 | Protein ycf2 |
| Mn_lnc-0086 | EXC28324.1 | hypothetical protein |
| Mn_lnc-0099 | EXC43199.1 | Legumin A |

(XBS)

**Table S6.** Summary of tissue specific genes in leaf with neighbor coding genes and functions.

| **LncRNA ID** | **Neighbor Coding Gene** | **Function** |
| --- | --- | --- |
| Mn_lnc-0021 | EXB50291.1 | hypothetical protein |
| Mn_lnc-0022 | EXB50426.1 | putative beta-1%2C3-galactosyltransferase 2 |
| Mn_lnc-0023 | EXB50920.1,  EXB50928.1,  EXB50929.1 | (-)-germacrene D synthase  (-)-germacrene D synthase  Pinene synthase |
| Mn_lnc-0027 | EXB53001.1 | Trafficking protein particle complex subunit 4 |
| Mn_lnc-0036 | EXB58188.1 | ,60S acidic ribosomal protein P0 |
| Mn_lnc-0038 | EXB65076.1 | Heparanase-like protein 2 |
| Mn_lnc-0044 | ,EXB72462.1 | Cation/H(+) antiporter 4 |
| Mn_lnc-0045 | EXB75948.1 | Wall-associated receptor kinase-like 20 |
| Mn_lnc-0047 | EXB80599.1 | Zinc finger protein MAGPIE |
| Mn_lnc-0058 | EXB96347.1 | hypothetical protein |
| Mn_lnc-0060 | EXB97676.1,  EXB97677.1 | 3-oxo-5-alpha-steroid 4-dehydrogenase 2;  3-oxo-5-alpha-steroid 4-dehydrogenase 2 |
| Mn_lnc-0074 | EXC21270.1 | hypothetical protein |
| Mn_lnc-0077 | EXC23523.1 | hypothetical protein |
| Mn_lnc-0081 | EXC25272.1 | hypothetical protein |
| Mn_lnc-0088 | EXC30765.1 | hypothetical protein |
| Mn_lnc-0089 | EXC30689.1 | hypothetical protein |
| Mn_lnc-0090 | EXC31504.1, | putative receptor-like protein kinase |
| Mn_lnc-0094 | EXC35008.1 | hypothetical protein |

(XBS)

**Table S7**. Summary of tissue specific genes in male flower with neighbor coding genes and functions.

| **LncRNA ID** | **Neighbor Coding Gene** | **Function** |
| --- | --- | --- |
| Mn_lnc-0006 | EXB28594.1,  EXB28598.1 | Protein PROLIFERA  DNA mismatch repair protein Msh6-1 |
| Mn_lnc-0007 | EXB29133.1 | DnAJ-like protein |
| Mn_lnc-0008 | EXB29871.1 | hypothetical protein |
| Mn_lnc-0013 | EXB38817.1 | hypothetical protein |
| Mn_lnc-0019 | EXB48303.1 | Puromycin-sensitive aminopeptidase |
| Mn_lnc-0048 | EXB81017.1 | Serine/threonine-protein phosphatase PP1 |
| Mn_lnc-0054 | EXB93909.1 | ,Protein lap1 |
| Mn_lnc-0061 | EXB97657.1 | Cysteine-rich receptor-like protein kinase 25 |
| Mn_lnc-0068 | EXC13800.1 | hypothetical protein; |
| Mn_lnc-0069 | EXC14254.1 | hypothetical protein; |
| Mn_lnc-0072 | EXC20310.1,  EXC20311.1,  EXC20313.1 | Phosphoenolpyruvate/phosphate translocator 2  tRNA (adenine(58)-N(1))-methyltransferase catalytic subunit Isocitrate dehydrogenase [NADP] |
| Mn_lnc-0079 | EXC24965.1 | GDSL esterase/lipase |
| Mn_lnc-0084 | EXC25828.1 | ,Katanin p60 ATPase-containing subunit |
| Mn_lnc-0092 | EXC32126.1 | Elongation factor 1-delta;,Cation/H(+) antiporter 15 |
| Mn_lnc-0095 | EXC35507.1 | Purple acid phosphatase 25 |

(XBS)
